# Effect of Combined MgO Expansive Agent and Rice Husk Ash on Deformation and Strength of Post-Cast Concrete

**DOI:** 10.3390/ma18122815

**Published:** 2025-06-16

**Authors:** Feifei Jiang, Yijiang Xing, Wencong Deng, Qi Wang, Jialei Wang, Zhongyang Mao

**Affiliations:** 1 School of Civil Engineering, Nantong Institute of Technology, Nantong 226000, China; 999620140019@just.edu.cn; 2 China State Construction Engineering (Macau) Co., Ltd., Macau 999078, China; xingyijiang@cohl.com (Y.X.); dengwencong@hotmail.com (W.D.); wangqi_2019@cohl.com (Q.W.); 3 College of Materials Science and Engineering, Nanjing Tech University, Nanjing 211800, China

**Keywords:** MgO expansion agent, post pouring strip, shrinkage cracks, tall buildings, rice husk ash

## Abstract

This study investigates the effects of the combined addition of MgO expansive agent (MEA) and rice husk ash (RHA) on the performance of concrete. Results show that MEA absorbs water and competes with superplasticizers for adsorption, reducing early-age fluidity. In the later stages, its reaction with RHA generates M-S-H gel, accelerating slump loss. At early ages (up to 7 days), due to the slow hydration of MEA and partial replacement of cement, fewer hydration products are formed. Additionally, the pozzolanic reaction of RHA has not yet developed, resulting in the low early strength of concrete. In the later stages, Mg(OH)_2_ fills pores and enhances compactness, while the pozzolanic reaction of RHA further optimizes the pore structure. The internal curing effect also provides the moisture needed for continued MEA hydration, significantly improving later-age strength. Moreover, in the post-cast strip of a tall building, the internal curing effect of RHA ensures the effective shrinkage compensation by MEA under low water-to-cement ratio conditions. The restraint provided by reinforcement enhances the pore-filling effect of Mg(OH)_2_, improving concrete compactness and crack resistance, ultimately boosting long-term strength and durability.

## 1. Introduction

With the increasing performance demands on concrete in modern construction, improving the durability, crack resistance, and strength of concrete has become a major focus of research. Cement concrete tends to shrink due to factors such as hydration heat [1] and drying shrinkage [2], leading to shrinkage cracks that compromise its long-term durability. This issue is particularly prominent in high-strength concrete with low water-to-binder ratios, where the risk of shrinkage cracking is significantly elevated [3,4,5,6].

In the foundation slab construction of tall buildings, post-cast strips serve as a critical structural measure to accommodate and release differential settlement. However, these strips are usually subjected to high-strength design requirements, low water-to-binder ratios, dense reinforcement, and severe external restraint. As a result, they experience significant autogenous and drying shrinkage, making them highly susceptible to early-age cracking [7]. Once cracks form, they not only reduce the structural integrity and water tightness, but also accelerate the ingress of harmful ions, leading to steel corrosion, durability degradation, and shortened service life [8,9,10,11].

Due to its advantages such as low water demand [12], stable hydration products [13,14], and controllable expansion behavior [15,16], MgO expansive agent (MEA) has been widely used for shrinkage compensation in concrete structures such as dams and airport pavements. However, some researchers have raised concerns regarding the potential reduction in compressive strength caused by the use of MEA. They argue that, while MEA hydration compensates for shrinkage through macroscopic expansion, it also increases the porosity of concrete, negatively impacting its strength. Wang [17] reported that replacing 8% of cement with MEA increased the porosity of concrete and reduced its compressive strength by 17.2%, 16.4%, and 14.6% at 3, 28, and 180 days, respectively. While such strength loss may be acceptable for structures with low strength requirements, such as dams, it is not tolerable in tall buildings, where concrete strength is a critical design parameter. Any reduction in strength could prevent the project from meeting acceptance standards, making this the first major limitation of MEA in high-rise construction.

Moreover, in large-volume high-strength concrete structures, moisture is rapidly consumed by cement hydration, and external curing water cannot effectively penetrate into the interior of the foundation slab. Under these conditions, whether MEA can continue to hydrate remains uncertain. Liu [18] found that curing humidity significantly affects MEA’s hydration and expansion behavior. When the relative humidity falls below 80%, the MEA hydration becomes insufficient, reducing its shrinkage-compensating ability and resulting in net shrinkage of the concrete. Therefore, enhancing the degree of MEA hydration represents the second key challenge for its application in tall buildings.

To address the dual challenges of strength reduction and the insufficient hydration of MEA in mass concrete, our team innovatively proposed the combined use of MEA and rice husk ash (RHA). RHA has a highly porous internal structure and strong water-retention capability. It gradually releases stored water over time, thereby increasing the internal humidity [19] and promoting the sustained hydration of MEA under sealed or low-permeability conditions. A comprehensive study was conducted through laboratory tests on workability, shrinkage deformation, internal relative humidity, and compressive strength to investigate the synergistic effects of MEA and RHA on concrete performance. In addition, field applications were carried out to verify the effectiveness of this material system under real engineering conditions. The results demonstrated that the combined use of MEA and RHA effectively mitigates shrinkage cracking while maintaining high strength, offering a practical and durable solution for post-cast segments in the foundations of tall buildings.

## 2. Materials and Experimental Methods

### 2.1. Raw Materials and Mix Proportions

Cement: PO 42.5 grade Portland cement produced by Macau Cement Factory (Macao, China).

Coarse aggregate: Crushed granite with a continuous gradation of 5–20 mm, apparent density of 2575 kg/m^3^.

Fine aggregate: River sand with a fineness modulus of 2.7.

MEA: High-activity MEA produced by Jiangsu Sobute New Materials Co., Ltd. (Nanjing, China), with an activity of 110 s measured by the citric acid method.

Water-reducing agent: Master Glenium SKY produced by Master Builders Solutions (Beachwood, OH, USA), a polycarboxylate-based high-performance water reducer.

RHA: Supplied by Shijiazhuang Xuhan New Materials Technology Co., Ltd. (Shijiazhuang, China), with a density of 2.21 g/cm^3^ and a specific surface area of 23.9 m^2^/g as measured by the BET method.

**Figure 1:**
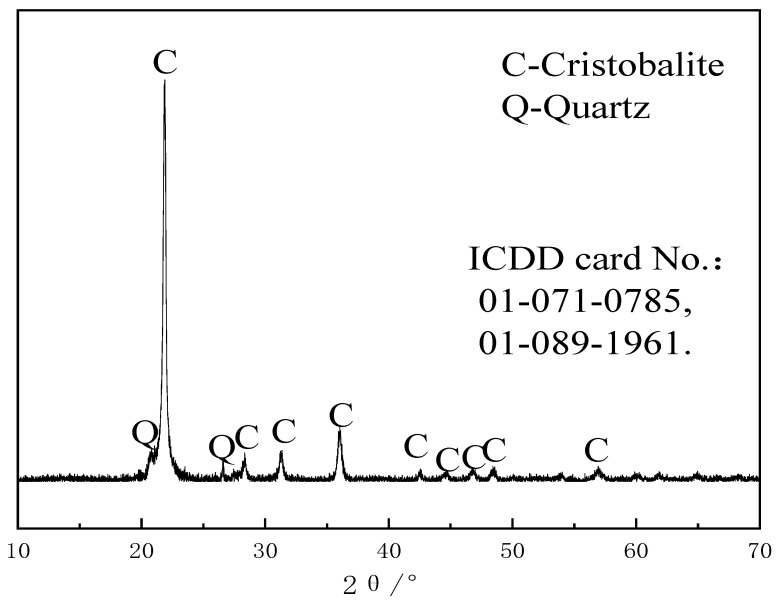
XRD pattern of rice husk ash.

The main chemical components of RHA are listed in Table 1, expressed in wt.%. And the SiO_2_ content in the rice husk ash is 78.25%. As shown in Figure 1, the XRD pattern of rice husk ash was obtained using a PANalytical X’Pert Pro MPD diffractometer produced by PANalytica (Eindhoven, Netherlands) with CuKα radiation, operating at 40 kV voltage and 40 mA current. The scan was conducted at a speed of 4°/min over a 2θ range of 5°–65°. The phase identification was performed using the PDF2-2004 ICDD database. SiO_2_ is primarily present in the form of cristobalite, with characteristic crystalline peaks appearing at 2θ = 21.98°, 28.44°, 31.46°, and 36.08°, corresponding to crystal planes 101, 111, 102, and 200, respectively. The crystallinity of the rice husk ash, calculated using Jade software (MDI Jade 6.0), is 47.73%.

The detailed concrete mix design is shown in Table 2. The initial concrete mix proportion was provided by the design institute and could not be modified at later stages. During the structural design phase, the required concrete strength was determined by the design institute, based on which the initial mix ratio was specified. This mix proportion was reviewed and confirmed by both the supervision unit and the material supplier, and therefore no changes were allowed. Consequently, the performance of the concrete was adjusted by incorporating MEA and RHA. To investigate the effects of different dosages of MEA and RHA on the performance of concrete, MEA and RHA were used as internal admixtures to replace cement by mass. The replacement ratios were 8% and 2%, corresponding to 36 kg and 9 kg, respectively [20]. The detailed concrete mix proportions are shown in Table 2, and four types of concrete were prepared: plain concrete (REF), concrete with 8% MEA (MC), concrete with 2% RHA (RC), and concrete with 8% MEA and 2% RHA (MR).

### 2.2. Experimental Methods

#### 2.2.1. Laboratory Tests

(1) Autogenous Deformation Test

The autogenous deformation test was conducted under constant temperature and sealed conditions. A VWS-15 vibrating wire strain gauge produced by Nanjing Genan Industrial Co., Ltd. (Nanjing, China) was used. The gauge has a gauge length of 150 mm and a strain measurement range from −1500 με to 1500 με. The deformation was calculated using Equation (1):

(1) ε=k∆F+(b−a)∆T

where ε is the autogenous deformation of concrete, 10^−6^/F; k is sensitivity of the strain gauge, 10^−6^/F; ΔF is the change in the measured value in real time relative to the base value of the vibrating modulus, F; b is temperature correction coefficient of the strain gauge, 10^−6^/°C; a is linear thermal expansion coefficient of concrete, 10^−6^/°C; ΔT is temperature change, °C.

**Figure 2:**
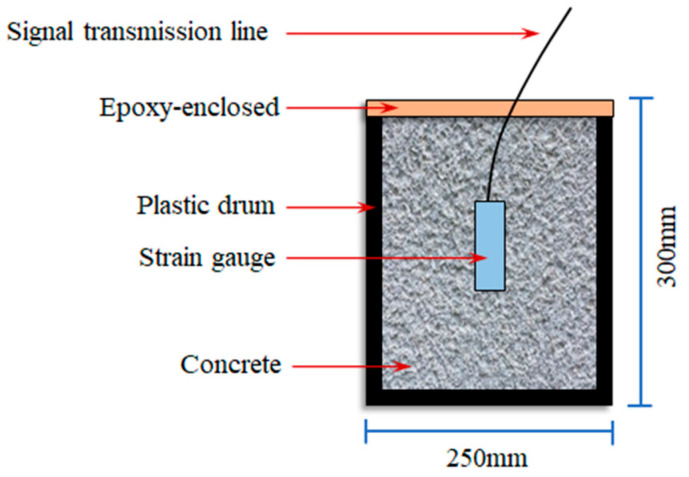
Autogenous deformation test of concrete.

PVC plastic buckets were used as casting molds, each with a diameter of 250 mm and a height of 300 mm. Freshly mixed concrete was poured into the buckets, compacted on a vibrating table until no air bubbles surfaced, then sealed with epoxy resin to prevent moisture exchange with the air. The sealed molds were then placed in a constant-temperature curing chamber at 20 °C. All casting and preparation were completed within 20 min. Finally, the strain of concrete with different mix proportions was recorded every 30 min to study its autogenous deformation. Detailed testing procedures are shown in Figure 2.

(2) Compressive Strength Test

The compressive strength test was conducted in accordance with the method described in GB/T 50081-2002 [21]. Cubic specimens with a side length of 150 mm were used, with three specimens cast for each type of sample and their average taken as the test result. When the concrete cubes reached the specified curing age, they were removed from curing, and surface moisture was wiped off with a towel before performing the compressive strength test. Three specimens were tested for each mix proportion, and the final result was taken as the average of the three specimens.

#### 2.2.2. On-Site In Situ Tests

(1) Project Overview

The construction project is a tall building located in Macau. The development includes six towers, each 149.90 m tall with 50 floors and an additional basement level. The site area is approximately 20,000 m^2^, with a total building area of about 330,000 m^2^. The foundation consists of pile caps supported by bored piles and prestressed concrete piles, which are then connected via a bottom slab. Large-diameter bored piles are used for the tower foundations, while the podium foundations use high-strength prestressed pipe piles.

Due to the heavy self-weight of the main tower, the foundation experiences significantly more settlement than the podium, and the bearing capacity of the reclaimed land foundation is relatively low. If left unaddressed, differential settlement between the main tower and the podium may lead to additional internal stresses, which can cause cracks, tilting, and affect the building’s safety and durability. Therefore, multiple post-cast settlement joints were installed between the main tower and the podium. The thickness of the post-cast joints is the same as that of the bottom slab, both being 1 m. Concrete is poured into the post-cast joint only after the differential settlement between the tower and podium has stabilized, thereby reducing the additional stress caused by settlement differences.

**Figure 3:**
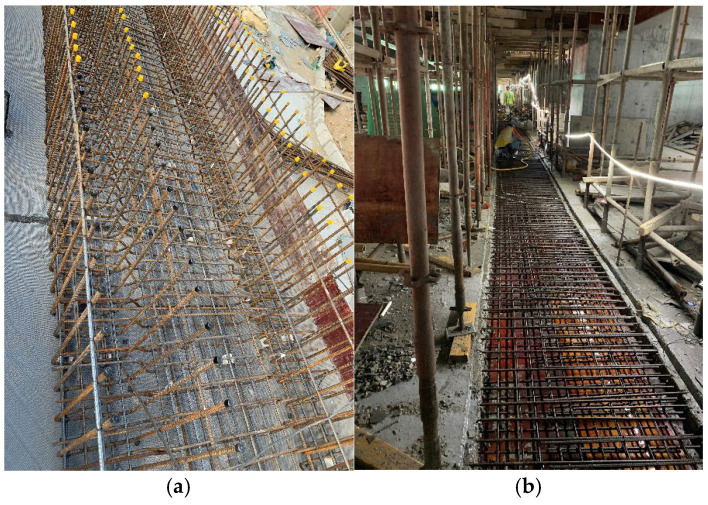
Construction of the settlement post-cast joint: (**a**) Rebar binding; (**b**) Formwork and rebar installation.

**Figure 4:**
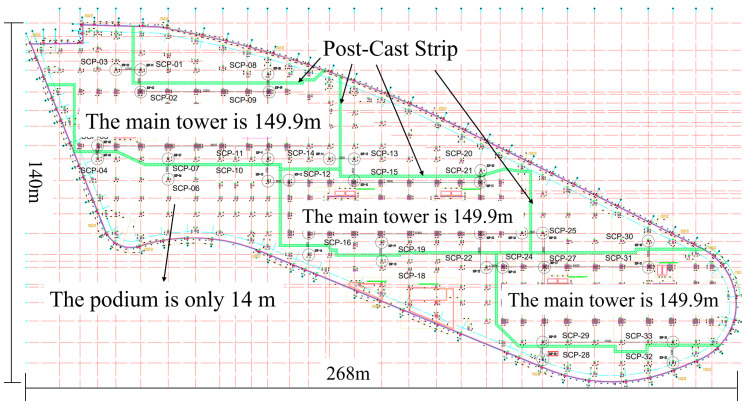
Schematic plan view of the post-cast strip layout.

**Figure 5:**
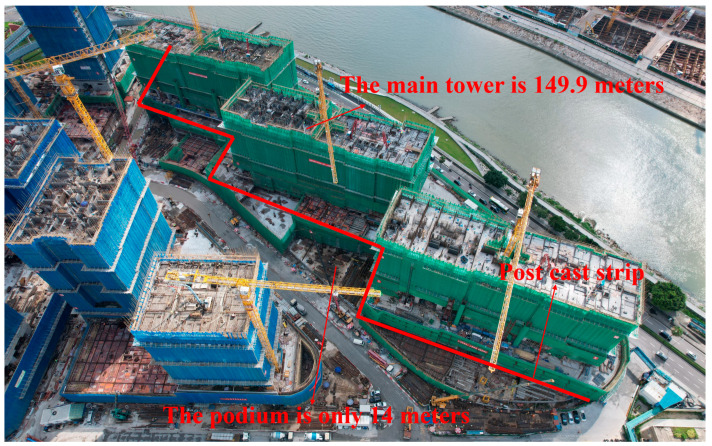
Top view of high-rise buildings.

As shown in Figure 3, the post-cast joint serves as an important structural measure for releasing settlement deformation and is typically located at the junction between the main tower and the podium. The plan layout of the post-cast strip is shown in Figure 4. The top view of the high-rise building is shown in Figure 5. However, due to the heat of hydration and moisture evaporation, the concrete in the post-cast joints tends to shrink, leading to several potential problems. Firstly, shrinkage-induced cracks compromise structural integrity, reduce durability, and may even affect the mechanical performance of the joint. Secondly, shrinkage cracks can form water channels. In reclaimed land environments with high groundwater levels, this increases the risk of water ingress and threatens structural safety. Finally, cracks may lead to reinforcement corrosion. To compensate for the shrinkage of the post-cast joint concrete, a composite mixture of 8% MEA and 2% RHA was used in this project. The specific mix proportions are shown in Table 2.

(2) Concrete Pouring

**Figure 6:**
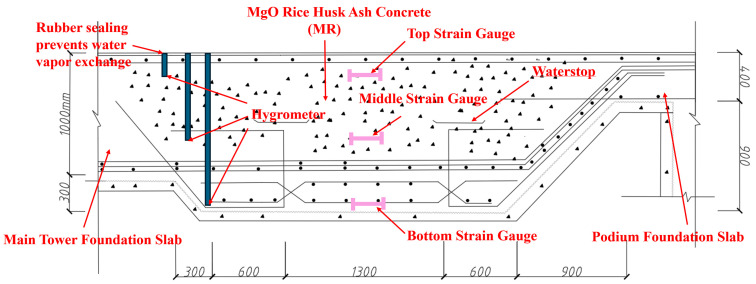
Post-cast concrete pouring and on-site testing.

As shown in Figure 6, in order to study the temperature, deformation, and humidity changes in the concrete, three strain gauges and hygrometers were embedded at different heights of the bottom slab. Strain Gauge No. 1 was placed near the surface (50 mm from the top surface) to measure the deformation of surface concrete. Strain Gauge No. 2 was embedded at the mid-depth (1/2 h = 650 mm) to measure the deformation in the center. Strain Gauge No. 3 was located near the bottom (50 mm from the bottom surface) to capture the deformation at the base.

As a comparison, a 150 cm concrete cube (without MEA and RHA) was cast and placed at ground level (to ensure it was in the same environmental conditions as the slab concrete with MEA). Strain Gauge No. 4 was used to measure its deformation. At the same positions, three hygrometers were also installed to monitor internal humidity changes in the concrete. The hygrometer was provided by Nanjing Genan Industrial Co., Ltd., with a measurement range of 10–100% RH and a sensitivity of 0.2% RH. The openings were sealed with rubber stoppers to prevent moisture exchange, ensuring accurate internal humidity measurements.

To assess the degree of MEA hydration under real construction conditions, three reserved holes with a diameter of 80 mm and depths of 50 mm, 650 mm, and 1250 mm were made during the mass concrete casting. These holes were used to place neat paste specimens containing MEA and RHA, in order to study the effects of actual field conditions (temperature and humidity) on MEA hydration.

(3) Evaluation of the Hydration Degree of MEA

Neat paste specimens containing MEA and RHA were placed into the reserved holes in the concrete slab. After reaching the specified curing age, approximately 50 mg of each sample was taken. Hydration was stopped by soaking the samples in absolute ethanol for 3 days. The samples were then dried in a vacuum oven at 80 °C and ground into powder using an agate mortar.

The degree of hydration of MEA was determined using differential thermal analysis (DTA). The heating rate was 10 °C/min, with a temperature range from 30 °C to 1000 °C. Nitrogen gas was used as the protective atmosphere during heating. The hydration degree of MgO was calculated using Equation (2) [22]:

(2) $$H_{MgO}=\frac{40\times {Massloss}_{∆T}}{D\times 18\times (1-{Massloss}_{950\;{}^{\circ}C})}$$

where $H_{MgO}$ is the degree of MgO hydration, %; ${Massloss}_{∆T}$ is the mass loss percentage corresponding to the endothermic peak of Mg(OH)_2_, %; ${Massloss}_{950\;{}^{\circ}C}$ is the mass loss percentage at 950 °C, %; and *D* is the dosage of MgO in the paste at the initial time, %.

(4) On-Site Strength Testing

During the post-casting concrete, standard concrete cube specimens (50 mm in side), without MEA and RHA, were cast simultaneously as control samples. To ensure the reliability of the data, three cubic specimens were cast, and their average value was taken as the compressive strength. At the specified curing ages, the compressive strength of the in-place concrete slab was tested using the rebound method, following the JGJ/T 23-2011 [23]. The compressive strength of the control cube specimens was tested according to the GBT 50107-2013 [24]. This approach was used to study the influence of MEA and RHA on concrete strength under actual construction conditions, in order to evaluate the feasibility of using MEA in mass concrete foundations.

(5) Pore Structure

Pore structure analysis was carried out using mercury intrusion porosimetry (MIP). For sample preparation, the neat paste specimens retrieved from the reserved holes in the slab were crushed in small fragments that were approximately 2 mm in size. These blocks were soaked in absolute ethanol for 24 h to terminate the hydration of cement and MEA. After ultrasonic cleaning, the samples were dried in a vacuum oven at 60 °C for 12 h and then sealed in airtight bags. For testing, two dried fragments were selected for MIP to analyze the pore structure, in order to assess the effects of MEA and RHA on the pore characteristics of the concrete.

## 3. Results and Discussion

### 3.1. Workability of Concrete

**Figure 7:**
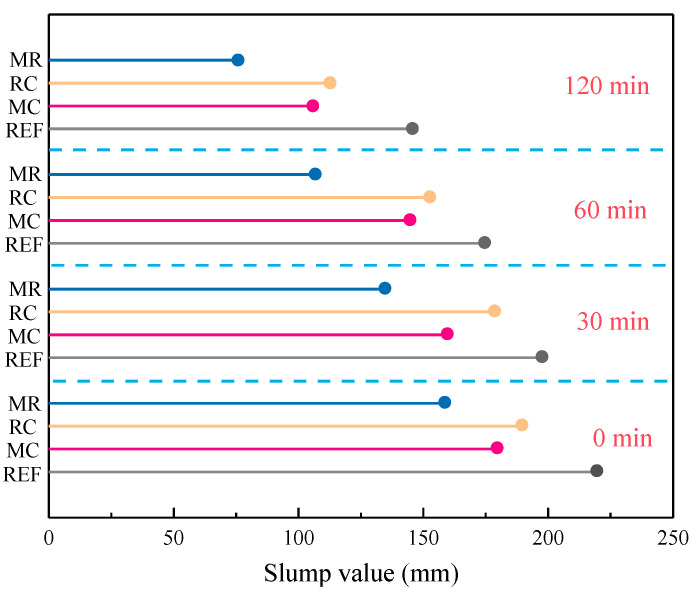
The effect of MEA and RHA on the workability of concrete.

The workability of concrete incorporating both MEA and RHA is shown in Figure 7. The addition of MEA significantly reduced the fluidity of the concrete. There are three main reasons for this effect at the early stage. First, the water absorption of MEA affects the wetting and dispersion of cement particles. MEA has a large specific surface area and strong hydrophilic properties. When the mixing water enters the paste, MEA will preferentially absorb part of the free water, reducing the amount of free water around the cement particles, which prevents the cement particles from being fully wetted and dispersed evenly [25]. Second, MEA particles release Mg^2+^ ions during hydration. These cations interact with the negatively charged surfaces of cement particles, reducing the electrostatic repulsion between the particles and making it easier for them to cluster together. The flocculation effect is enhanced, and the flowability of the cement particles decreases, leading to the higher viscosity of the paste [26]. Third, MEA particles have a certain adsorptive capacity and will compete with cement particles for the adsorption of polycarboxylate superplasticizers, reducing the effective concentration of the superplasticizer on the cement surface and weakening the dispersion effect [27].

In the later stages of hydration, MEA forms Mg(OH)_2_, which interacts with other hydration products such as C-S-H gel to form a network structure. This increases the yield stress of the paste and further reduces fluidity [28]. Since the hydration rate of MEA is slower than that of cement, the viscosity effect of Mg(OH)_2_ is not significant at early stages. However, as the hydration rate of MEA increases, its negative impact on flowability becomes more pronounced, which is the main reason for the significant slump loss observed after 120 min in MEA-containing concrete.

When both MEA and RHA are used together, the SiO_2_ in RHA can react with Mg(OH)_2_ in the alkaline environment to form M-S-H gel through a pozzolanic reaction. The reaction equation is as follows:

SiO_2_ + Mg(OH)_2_→M-S-H (hydrated magnesium silicate) (3)

The formation of M-S-H gel further reduces the free water, causing the paste to gradually thicken over time and accelerating the slump loss. This leads to a reduction in slump to 76 mm at 120 min, making pumping difficult. Therefore, from a construction perspective, the composite concrete should be completed within 120 min after mixing (including the total time for transportation and pouring).

### 3.2. Deformation Performance of Concrete

**Figure 8:**
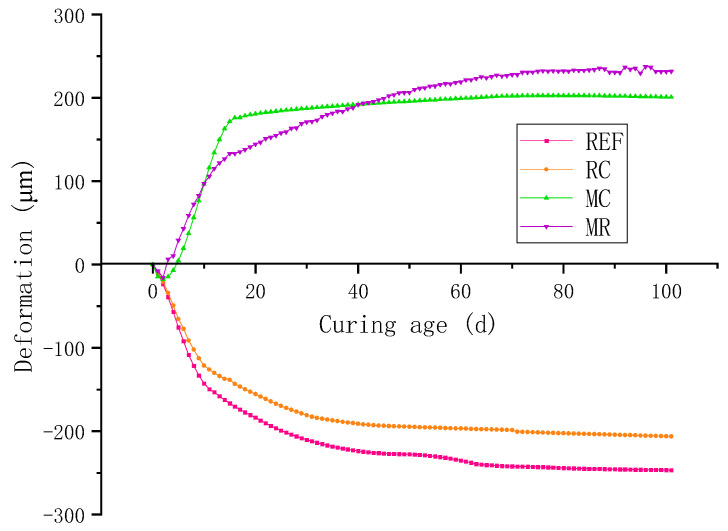
Deformation of concrete at different ages.

Figure 8 illustrates the deformation performance of concrete at different ages. It can be observed that ordinary concrete undergoes rapid shrinkage during the early stage (0–12 days). This shrinkage is primarily due to the reaction between cement and water, which produces hydration products with reduced volume. Since the hydration reaction occurs very quickly at early ages, the shrinkage increases rapidly, reaching −153 με at 12 days.

Subsequently, the cement hydration reaction slows down, and the shrinkage rate of the concrete decreases significantly. By 80 days, the shrinkage gradually increases to −244 με. After 80 days, the cement hydration is essentially complete, the shrinkage rate significantly decreases, and the shrinkage tends to stabilize, showing only slight fluctuations, eventually stabilizing at around −248 με. When such shrinkage is restrained, tensile stress develops inside the concrete, which is a major cause of early-age cracking in high-strength concrete [29].

With the addition of RHA alone, shrinkage is reduced, and the time for shrinkage to stabilize is shortened from 80 days to about 70 days. The first reason is that RHA is rich in SiO_2_, which undergoes a secondary hydration reaction with Ca(OH)_2_ to form the C-S-H gel, improving the compactness of the concrete and its resistance to shrinkage. The second reason is that the porous structure of RHA enables moisture redistribution. When free water is consumed, a relative humidity gradient forms inside the concrete, inducing RHA to release moisture, which effectively reduces autogenous shrinkage. Additionally, due to the internal curing effect of RHA, the later hydration of cement is accelerated, thus shortening the overall shrinkage period [30].

When 8% MEA is added alone, the expansive behavior of MEA compensates for the shrinkage of the concrete. However, due to the intense early hydration of cement, significant early-age shrinkage still occurs, and since MEA hydrates more slowly than cement, a shrinkage of −15 με is observed at 0–2 days. As MEA gradually hydrates and expands, the overall deformation of the concrete shifts from shrinkage to expansion. At 15 days, the volume rapidly increases to 176 με. Thereafter, the expansion rate significantly decreases, and by 75 days, the concrete slowly expands to 201 με. After that, the expansion and shrinkage of the concrete essentially reach equilibrium, and the volume remains stable with no significant fluctuations [12,31].

When both 8% MEA and 2% RHA are added, the pozzolanic reaction of RHA consumes part of the calcium hydroxide, lowering the alkalinity of the pore solution and thus suppressing the hydration rate of MEA [12]. As a result, the early expansion is lower than that of the MEA-only mix. However, in the later stage, RHA’s internal curing effect provides additional water for MEA hydration, leading to increased expansion. At 100 days, the expansion is 14.9% greater than that of the MEA-only mix, reaching 231 με.

### 3.3. Mechanical Properties of Concrete

**Figure 9:**
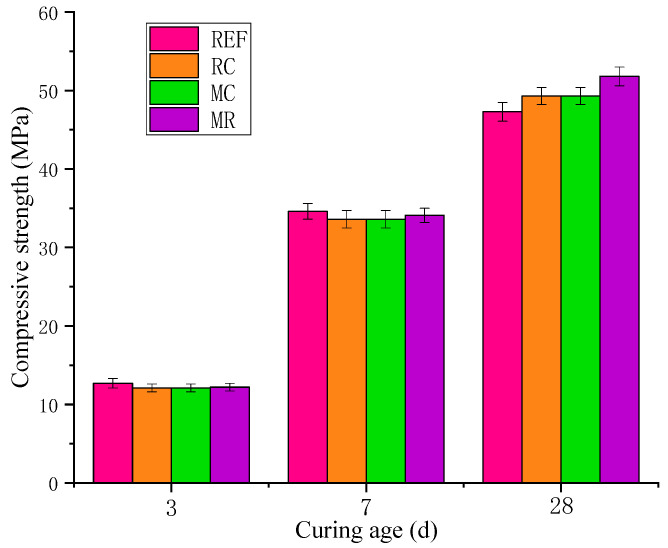
Compressive strength of concrete at different ages.

The compressive strength of concrete at various curing ages is shown in Figure 9. In the early stages (3 and 7 days), the partial replacement of cement with MEA results in a reduction in the formation of cement hydration products (such as C-S-H gel), thereby decreasing the early strength. Moreover, the hydration rate of MEA is significantly slower than that of cement, and it does not sufficiently hydrate in the early stages to contribute to strength development, further limiting the early strength gain. As a result, the compressive strength of the MEA-containing concrete (MC) is 9.4% and 4.3% lower than that of the reference mix (REF) at 3 and 7 days, respectively. As the curing time increases, MEA gradually hydrates to form Mg(OH)_2_, which precipitates and fills the pores within the concrete. This reduces porosity and enhances the compactness of the cement paste, thereby improving the later-age strength. By 28 days, the compressive strength of the MC mix exceeds that of REF by 2.7%.

In ordinary cement systems, early strength primarily comes from hydration products such as C-S-H gel and Ca(OH)_2_. However, RHA itself lacks early hydration activity and does not directly produce strength-contributing substances, leading to reduced early strength [32]. Consequently, the 3-day and 7-day compressive strengths of the RHA-containing mix (RC) are 4.7% and 2.9% lower than those of the reference mix (REF), respectively. As the curing age increases, cement hydration gradually releases Ca(OH)_2_, which accelerates the pozzolanic reaction of RHA. This reaction forms additional C-S-H gel, improving the density and strength of the cement paste. In addition, RHA particles can act as nucleation sites to promote a more uniform distribution of C-S-H gel, reduce large pore structures, and enhance later-age strength [33]. By 28 days, the strength of the RC mix exceeds that of REF by 4.2%.

**Figure 10:**
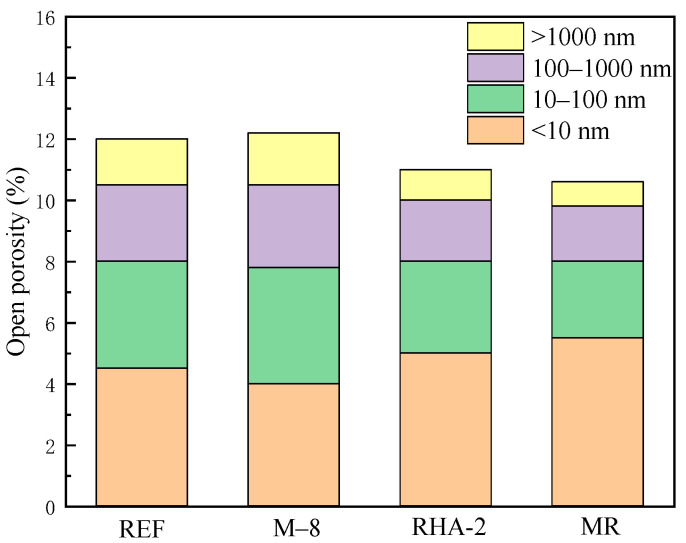
Pore structure of concrete at 28 days.

When both MEA and RHA are used together, in the early stages (3 and 7 days), both the pozzolanic reaction of RHA and the hydration of MEA proceed relatively slowly, resulting in limited contributions to early strength. At 3 days, the compressive strength is 3.9% lower than REF. However, in the later stage (28 days), as the concrete continues to harden, cement hydration consumes water and causes internal drying. RHA, with its water storage capability, gradually releases moisture during this period [34], providing a favorable environment for continued MEA hydration, thus promoting the formation of Mg(OH)_2_. Moreover, Mg(OH)_2_ can react with SiO_2_ in RHA to form magnesium silicate hydrate (M-S-H) [35]. The formation of M-S-H gel further optimizes the pore structure, enhances the compactness of the paste, reduces porosity, and significantly improves the long-term strength and durability of the concrete. As shown in Figure 10, it is precisely the synergistic effect of RHA and MEA that reduces the porosity and increases the density of the concrete, resulting in a 28-day compressive strength that is 9.5% higher than that of REF.

### 3.4. Field Test Results and Analysis

(1) Engineering Challenges of Post-Cast Concrete in Tall buildings

The post-cast concrete segments in tall buildings present several significant engineering challenges that necessitate advanced material solutions. First, these segments are massive in both area and thickness, leading to considerable temperature drop shrinkage and drying shrinkage after casting, which significantly increases the risk of shrinkage-induced cracking.

Second, the load conditions are highly complex. The post-cast segment must withstand a combination of static loads from the upper structure and dynamic loads such as vehicular traffic, thereby imposing stringent requirements on the compressive strength of the concrete.

Third, the project is located in a coastal reclamation area with a high groundwater level. As a critical part of the foundation system, the post-cast segment must provide excellent waterproofing to prevent groundwater infiltration and ensure the long-term safety of the structure.

Fourth, the durability requirements are exceptionally high. Given the extended service life of tall buildings, the foundation concrete must possess excellent durability and crack resistance to withstand long-term mechanical and environmental stresses.

Fifth, the unique geological conditions pose additional structural demands. The project is situated in a reclaimed area with relatively soft foundation soil. Due to the significant difference in self-weight between the main tower and the podium, the post-cast segment experiences substantial bending moments after differential settlement. As a result, it is essential to address not only shrinkage issues but also the strength demands imposed by this complex stress state.

Lastly, high workability is required. As shown in Figure 3, dense reinforcement is arranged in the post-cast segment to resist the heavy load, resulting in very narrow rebar spacing. This hinders concrete flow and makes workability a critical property during construction.

In summary, the post-cast concrete in this project must simultaneously meet strict requirements for strength, workability, and shrinkage crack resistance under challenging engineering and environmental conditions. These factors underscore the necessity of employing an optimized material system—such as the combined use of MEA and RHA—to ensure both performance and durability.

(2) Measured Temperature of Concrete Slab

**Figure 11:**
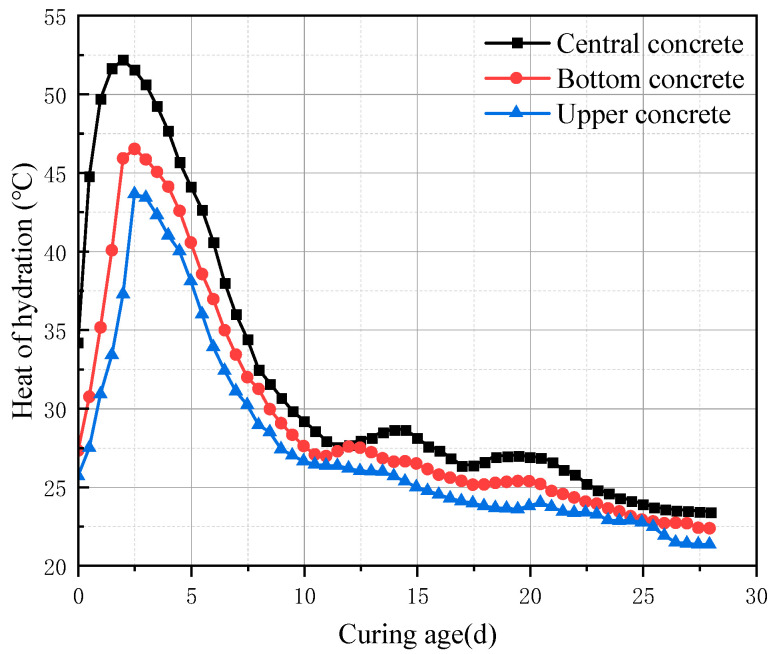
Measured temperatures of concrete at different locations.

The temperature variation of the post-casting concrete after casting is shown in Figure 11. In the early stage, due to the rapid hydration and exothermic reaction of cement, the temperature rose sharply, reaching its peak at 2 days. Owing to the poor thermal conductivity of concrete and the large thickness of the slab, heat in the core was difficult to dissipate, with the maximum core temperature reaching 52.2 °C. In contrast, the surface layer could exchange heat with the atmosphere more efficiently, resulting in a faster heat dissipation and a peak temperature about 20% lower than the core, at 43.6 °C.

In the middle stage, as cement continued to be consumed, the rate of heat release from hydration decreased, and the temperature dropped rapidly to 24 °C by day 15. At this point, thermal shrinkage led to significant volume contraction. Under the combined restraint of the foundation, formwork, and the early-cooled surface concrete, substantial tensile stress developed inside the concrete, which is one of the main causes of cracking in mass concrete [36].

In the later stage, as the hydration reactions of the cementitious materials were essentially completed, no further heat was generated. The slab temperature gradually aligned with the ambient temperature, fluctuating slightly in response to environmental changes.

(3) Measured Deformation of the Concrete Slab

**Figure 12:**
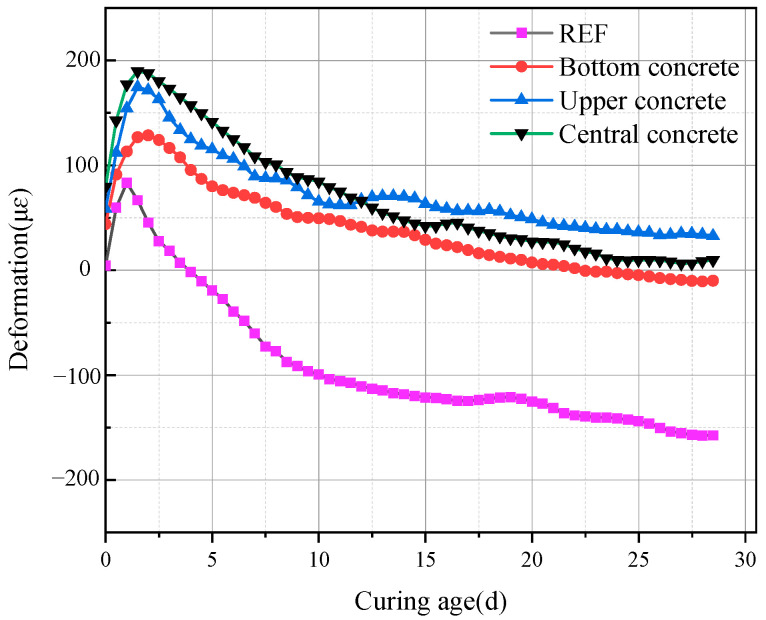
Measured deformation of concrete at different locations.

Figure 12 presents the measured deformation of concrete at different heights. In ordinary concrete, the volume continuously decreased after casting, with a shrinkage of 75 με observed by day 10. After adding the expansive agent, the MEA-induced expansion compensated for the shrinkage, and all three monitored positions exhibited varying degrees of expansion.

At the core, due to higher temperatures, the hydration rate of MEA was greater, resulting in significant early expansion. However, since the concrete had not yet fully hardened at this stage, part of the expansion was ineffective, leading to lower total long-term expansion at the core compared to the surface. At the surface, early temperature and hydration rate were lower; yet in the later stage, water supplementation through mist spraying promoted the sustained hydration of MEA, resulting in greater expansion than that observed at the core.

(4) Relative Humidity Inside Concrete at Different Positions

**Figure 13:**
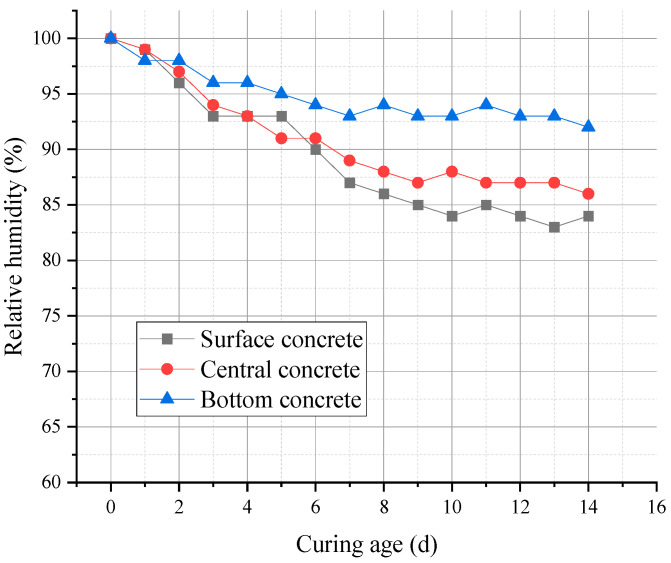
Relative humidity inside concrete at different locations.

Figure 13 illustrates the internal relative humidity of concrete at different locations within the post-cast strip. After casting, hydration gradually consumed internal water, leading to a drop in internal humidity. The surface concrete, benefiting from external water curing, experienced a slower humidity decline, maintaining over 92% relative humidity even at day 15.

Due to the large thickness of the post-cast strip, external water has difficulty penetrating into the middle and lower sections. Moreover, the accumulation of hydration heat raises the temperature, causing intense cement hydration and rapid consumption of moisture, which results in a faster decline in humidity in the core and bottom areas [37]. RHA (rice husk ash), with its porous structure and internal curing effect, helped mitigate this moisture loss and promoted the continued hydration of both cement and MEA. As shown in Figure 13, the relative humidity in the core and bottom regions remained above 84% during the 15-day period, indicating that RHA effectively enhanced the moisture retention capability of the concrete.

The combination of RHA and MEA not only prevented shrinkage cracking in mass concrete but also supported the long-term hydration of MEA. The addition of RHA ensured that even under conditions of a low water-to-binder ratio and reduced humidity, MEA could still hydrate sufficiently and exhibit favorable expansion performance, thereby improving the long-term stability and strength of the concrete.

(5) Degree of MEA Hydration

**Figure 14:**
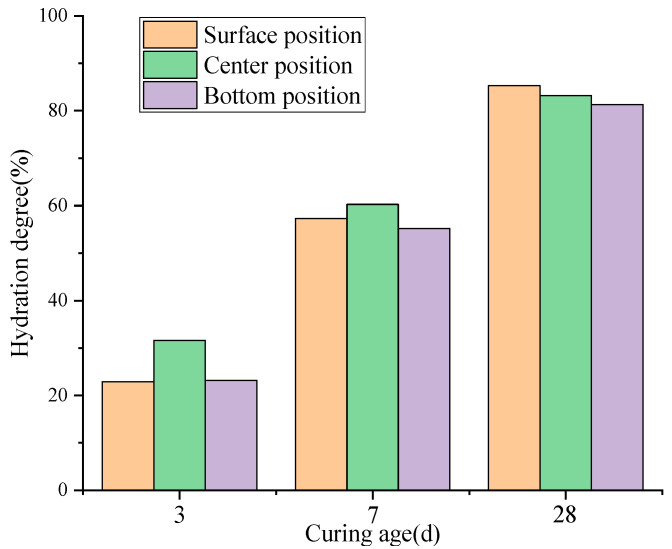
Hydration degree of MEA at different locations.

The measured hydration degree of MEA at different locations of the concrete slab after on-site sampling is shown in Figure 14. After 3 d of casting, the hydration degree of MEA in the central region was 31.6%, which was 26.9% higher than that in the surface region (22.9%). This difference is primarily due to the higher temperature at the slab center, as MEA is highly sensitive to temperature and elevated temperatures can significantly accelerate its hydration [38].

At 7 d, the degree of MEA hydration in the central region was 60.3%, which was 5.2% higher than that in the surface region (57.3%). The difference between the two was significantly reduced compared to that at 3 d, mainly because the temperature had decreased, leading to a reduced MEA hydration rate in the core region. Subsequently, as the temperature further decreased and moisture was consumed, the MEA hydration rate in the central region slowed down markedly, further narrowing the gap with the surface region.

At 28 d, the MEA hydration degree in the surface region reached 85.3%, which was 2.5% higher than that in the central region. This was because, as the hydration of cement and MEA progressed, the free water in the core was gradually consumed, and external moisture could not penetrate into the central area. The resulting decline in humidity led to a lower MEA hydration rate. In contrast, the surface region received a continuous water supply due to curing, so its hydration rate during the later stage (7–28 days) was higher than that of the central region. The MEA at the bottom surface had a lower temperature than the core and lower humidity than the upper surface, so its hydration degree at 28 days was the lowest.

As shown in Figure 14, for the MEA hydration in the mass concrete floor slab, the early stage (0–3 d) was mainly controlled by temperature, while the middle and later stages (7–28 d) were mainly controlled by humidity. Due to the large dimensions of the floor slab in this high-rise building, with a maximum thickness of 1.3 m and a large amount of cement used, the heat of hydration was not easily dissipated, resulting in higher internal temperatures in the concrete. The higher the temperature, the greater the MEA hydration rate. At 3 days, the MEA hydration degree in different regions was all greater than 55%. In the later stages, due to the internal curing effect of rice husk ash, additional moisture was provided for MEA hydration, and the MEA hydration degree was greater than 80% in all regions.

(6) Measured Compressive Strength of Post-pour Concrete

**Figure 15:**
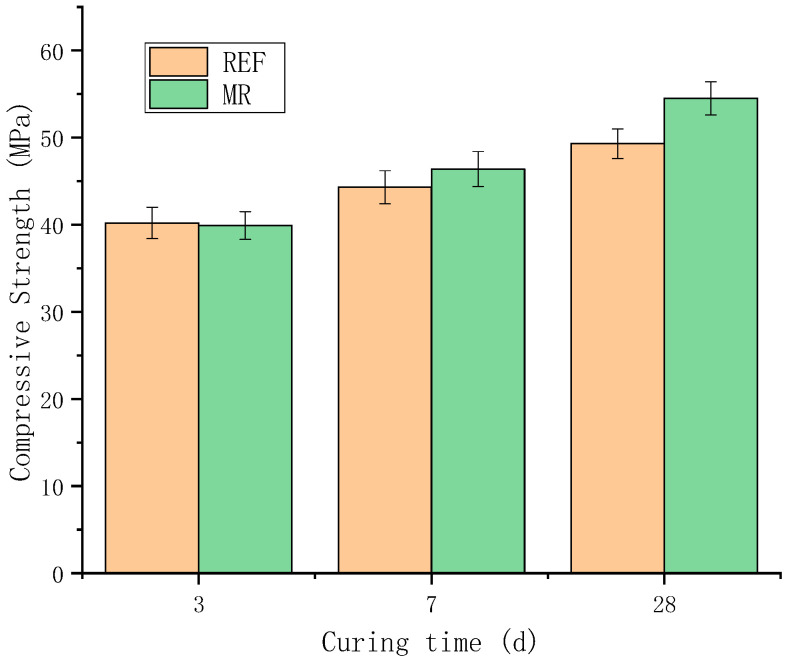
Compressive strength of concrete.

Figure 15 shows the measured compressive strength of the post-pour concrete at different curing ages. The concrete strength increased with curing time. At early ages (3 days), the strength of MR concrete was similar to that of ordinary concrete. In the later stages, as MEA continued to hydrate and expand, the growth of magnesium hydroxide in the restricted pore size under the reinforcement restraint increased the density, and the strength gradually exceeded that of ordinary concrete. At 28 days, the strength was 10.5% higher than that of ordinary concrete. Based on the results of both deformation and strength tests, it can be concluded that the restrained expansion of MEA effectively mitigates shrinkage cracking and improves the concrete strength in high-strength-reinforced concrete for high-rise buildings.

## 4. Conclusions

(1) The combined incorporation of MgO expansive agent (MEA) and rice husk ash (RHA) has demonstrated a synergistic effect on improving the crack resistance and durability of post-cast concrete used in tall building foundations. Their interaction effectively addresses both early-age shrinkage and long-term strength development.
(2) MEA provided expansion to counteract shrinkage; however, its hydration was limited in low-humidity, highly restrained environments. RHA, serving as an internal curing agent, improved internal moisture conditions, promoted the continuous hydration of MEA, and participated in pozzolanic reactions, forming additional C-S-H and M-S-H gels. This dual mechanism optimized the pore structure, enhanced matrix densification, and significantly reduced cracking risks.
(3) The composite mix showed reduced early-age workability due to the water absorption and ion exchange of MEA and the thickening effect of RHA. Slump loss was accelerated, and it is recommended that concrete be placed within 120 min of mixing to ensure good workability.
(4) Compared to conventional concrete, the composite concrete exhibited 10.5% higher 28 d compressive strength, while maintaining excellent shrinkage control. Field application demonstrated its suitability for high-strength, densely reinforced post-cast strips in tall buildings, offering a practical and effective approach to mitigating shrinkage-induced cracking in large-volume structural elements.

## Figures and Tables

**Table 1:** Chemical composition of rice husk ash/%.

Chemicals	SiO_2_	Al_2_O_3_	Fe_2_O_3_	CaO	MgO	SO_3_	Na_2_O	K_2_O	Cl	LOI
RHA	78.25	0.33	0.28	0.933	0.395	0.143	0.053	0.052	0.016	19.548

**Table 2:** Concrete mix design/kg·m^−3^.

No.	Cement	Coarse Aggregate	Fine Aggregate	Water	Water Reducer	MEA	RHA
REF	450	980	740	172	5.7		
MC	414	980	740	172	5.7	36	
RC	441	980	740	172	5.7		9
MR	405	980	740	172	5.7	36	9

## Data Availability

The original contributions presented in this study are included in the article. Further inquiries can be directed to the corresponding authors.
